# The capacity for multistability in small gene regulatory networks

**DOI:** 10.1186/1752-0509-3-96

**Published:** 2009-09-21

**Authors:** Dan Siegal-Gaskins, Erich Grotewold, Gregory D Smith

**Affiliations:** 1 Mathematical Bioscience Institute, The Ohio State University, Columbus, OH 43210, USA; 2 Department of Plant Cellular and Molecular Biology and Plant Biotechnology Center, The Ohio State University, Columbus, OH 43210, USA; 3 Department of Applied Science, The College of William and Mary, Williamsburg, VA 23187, USA

## Abstract

**Background:**

Recent years have seen a dramatic increase in the use of mathematical modeling to gain insight into gene regulatory network behavior across many different organisms. In particular, there has been considerable interest in using mathematical tools to understand how multistable regulatory networks may contribute to developmental processes such as cell fate determination. Indeed, such a network may subserve the formation of unicellular leaf hairs (trichomes) in the model plant *Arabidopsis thaliana*.

**Results:**

In order to investigate the capacity of small gene regulatory networks to generate multiple equilibria, we present a chemical reaction network (CRN)-based modeling formalism and describe a number of methods for CRN analysis in a parameter-free context. These methods are compared and applied to a full set of one-component subnetworks, as well as a large random sample from 40,680 similarly constructed two-component subnetworks. We find that positive feedback and cooperativity mediated by transcription factor (TF) dimerization is a requirement for one-component subnetwork bistability. For subnetworks with two components, the presence of these processes increases the probability that a randomly sampled subnetwork will exhibit multiple equilibria, although we find several examples of bistable two-component subnetworks that do not involve cooperative TF-promoter binding. In the specific case of epidermal differentiation in *Arabidopsis*, dimerization of the GL3-GL1 complex and cooperative sequential binding of GL3-GL1 to the CPC promoter are each independently sufficient for bistability.

**Conclusion:**

Computational methods utilizing CRN-specific theorems to rule out bistability in small gene regulatory networks are far superior to techniques generally applicable to deterministic ODE systems. Using these methods to conduct an unbiased survey of parameter-free deterministic models of small networks, and the *Arabidopsis *epidermal cell differentiation subnetwork in particular, we illustrate how future experimental research may be guided by network structure analysis.

## Background

The availability of high-throughput techniques for gene expression analysis and identification of promoter-transcription factor (TF) interactions has led to characterization of the intricate gene regulatory networks that govern organism behavior [1-3]. These networks are composed of a large number of small and topologically distinct subnetworks, including the overrepresented 'network motifs' [4-7]. In recent years, dynamical systems modeling of regulatory and signaling pathways has provided insight into the equilibrium states and transient dynamics of such subnetworks [8,9]; for example, detailed cellular and subcellular models demonstrate that interconnected positive and negative feedback loops may give rise to the phenomena of oscillations, excitability, and the existence of multiple stable equilibria (e.g., bistability) [10,11].

Bistability in particular is ubiquitous in biological systems ranging from biochemical networks to ecosystems [12-16]. In bistable systems, graded inputs (e.g., the concentration of a specific hormone) are converted into a discontinuous ON/OFF response [17-20]. Switch-like behavior is also a characteristic of many developmental processes, and it has been suggested that the maintenance of two distinct phenotypic states in the absence of genetic or environmental differences may sometimes be attributed to bistability in an underlying gene network [21].

**Figure 1:**
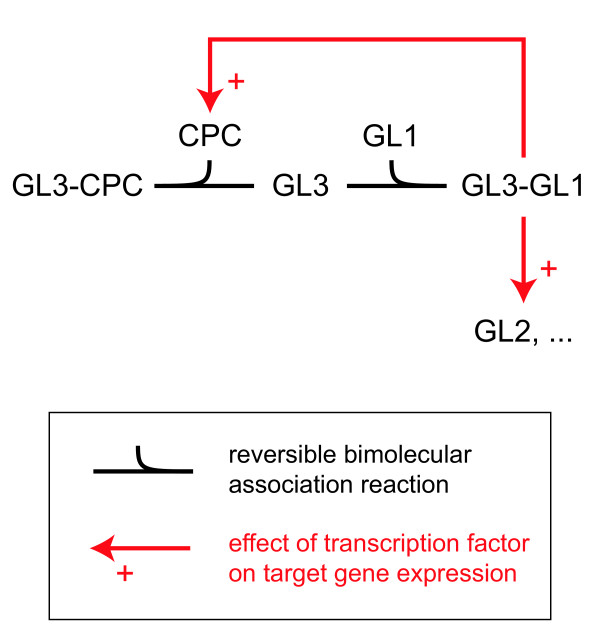
**Arabidopsis trichome differentiation network**. In *Arabidopsis *the network responsible for the differentiation of trichomes from pluripotent epidermal cells consists of a well-defined group of regulatory proteins belonging to the R2R3-MYB (e.g., GL1) and basic helix-loop-helix (bHLH) (e.g., GL3) classes. GL1 proteins complex with GL3 proteins to directly regulate other transcription factors (e.g., GL2) that positively induce trichome initiation, as well as small inhibitory proteins (e.g., CPC). The interaction of GL3 with CPC or any of the other small MYB proteins such as TRY, ETC1, ETC2 or MYBL2 prevent GL3 from interacting with GL1 [24], thus creating a non-functional complex. In this simplified diagram, CPC represent all of these small MYB proteins which clearly show overlapping functions.

An intriguing system that exhibits phenotypic bistable behavior, and as such is an excellent candidate for the study of the potential role of bistability in cell fate determination, is the formation of unicellular leaf hairs (trichomes) in the model plant *Arabidopsis thaliana*. In *Arabidopsis*, trichomes differentiate from pluripotent epidermal cells by the action of regulatory proteins belonging to the R2R3-MYB (e.g., GL1) and basic helix-loop-helix (bHLH) (e.g., GL3) classes [22]. These positive regulators directly regulate other TFs (e.g., GL2) that positively induce trichome initiation, as well as small inhibitory proteins (e.g., CPC). A simplified version of this network is shown in Fig. 1. An important aspect of trichome differentiation not indicated in Fig. 1 is the free movement of inhibitory proteins to adjacent epidermal cells [23] where they prevent GL3 from interacting with GL1 [24], thus creating a domain of surrounding cells that will not become trichomes and resulting in a characteristic spatial pattern.

Previous attempts at modeling this cell fate determination system have aimed at explaining how trichome patterns form out of a field of initially equivalent epidermal cells, but have ignored the question of how the primary identity decision is made [25,26]. Such models assume an underlying mechanism of either the 'activator-inhibitor' or 'trapping/depletion' type, both of which include positive regulation of GL3 by the GL3-GL1 active complex. Consistent with the activator-inhibitor model [27], it has been shown experimentally that the activators do positively control the diffusible inhibitors [3]. However, although recent work has suggested that a positive feedback loop may be involved in cell fate specification in the root epidermis [28], no such direct positive feedback has been found in the leaf. Indeed, the regulators of trichome patterning in the leaf may in fact be involved in a negative feedback loop, as recently demonstrated for GL3 [29]. This poses the question of whether the regulatory subnetworks known to be involved in trichome initiation have the capacity for bistability, or whether this is unlikely given the absence of experimental evidence for direct positive autoregulation.

### Tools for establishing multistability in ODE models of regulatory networks

Determining if a given gene regulatory network has multiple equilibria first requires the construction of an appropriate mathematical model of the system. We consider deterministic ODE models that take the form [30,31]

(1)

where *c *= (*c*_1_,⋯,*c*_*N*_)^*T *^is a vector of concentrations of *N *species and *F*(*c*) = (*F*_1_(*c*),⋯,*F*_*N*_(*c*))^*T *^is a continuous and differentiable matrix function that gives the rate of change of the concentrations over time. In this context, a biologically meaningful equilibrium solution of Eq. 1 is a set of (nonnegative) concentrations leading to *dc*/*dt *= 0, that is, a vector *c*_*ss *_that satisfies *F*(*c*_*ss*_) = 0. Thus, a network with multiple equilibria is one with two or more distinct vectors *c *for which Eq. 1 yields *dc*/*dt *= 0, A given equilibrium is *stable *(i.e., persists after small perturbations) provided the Jacobian matrix,

(2)

has *N *eigenvalues with negative real part when evaluated at the equilibrium concentrations. Since in practice there is poor knowledge of the rate constants and binding affinities that occur in *F *(*c*), we are primarily interested in assessing a network's capacity for multiple equilibria or bistability without specifying these parameters, though all rate constants are assumed to be positive. A simple parameter-free graphical representation of the system is given in its *interaction graph *in which each species is represented as a graph node. A directed positive edge is drawn from species *i *to *j *if ∂*F*_*j*_/∂*c*_*i*_(*c*) > 0, a directed negative edge is drawn if ∂*F*_*j*_/∂*c*_*i*_(*c*) < 0. No edge is drawn if ∂*F*_*i*_/∂*c*_*i*_(*c*) = 0.

**Figure 2:**
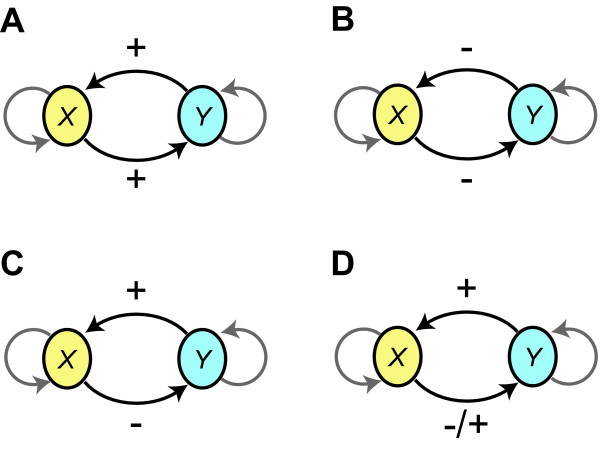
**Circuits in interaction graphs**. A circuit in the interaction graph is a closed sequence of oriented edges in which no edge is traversed more than once. In the figure shown here, there exists a circuit from species X to species Y (along the lower edge) and back to X (along the upper edge). The sign of the circuit is the product of the signs of the included edges, and can be positive (A and B), negative (C), or variable (D), in which case the sign changes with the circuit's location in phase space. Self-loops, which can also be positive or negative, are shown in gray.

As originally conjectured by Thomas [32] and later proven [33], a necessary condition for multiple equilibria (also known as multistationarity) in Eq. 1 is the presence of a positive *circuit *in the interaction graph (i.e., a circuit for which the product of the signs of the edges is positive; see Fig. 2) for at least one set of species concentrations. Thus, the absence of any positive circuits in a network rules out the capacity for bistability, regardless of the parameters chosen for the model. Another necessary condition for multistationarity proposed by Kaufman [34] concerns the *nuclei *of the interaction graph, which are defined as unions of one or more disjoint circuits involving all the vertices of the interaction graph. Multistationarity requires either (i) the presence of a variable nucleus (that is, a nucleus with at least one edge displaying more than one sign depending on species concentration), or (ii) the presence of two nuclei of opposite signs, where the sign of a nucleus with *p *positive circuits is (-1)^*p*+1 ^[35]. Networks that do not satisfy either of these criteria may also be eliminated in a search for bistability.

When deterministic ODE models (Eq. 1) are constructed from specified elementary processes under the assumption of mass-action kinetics, their governing equations takes the special form of a *chemical reaction network *(CRN) [36],

(3)

where *S *is a stoichiometric matrix indicating the change in the number of molecules of each species in a particular reaction, and *ϕ*(*c*) is a vector of the reaction rates (written as polynomial functions of the species concentrations [37]). While both Eq. 1 and Eq. 3 are nonlinear ODEs, the specific form of CRNs enables the application of powerful CRN-specific network analysis techniques. One computational method can rule out multiple equilibria in a CRN by determining if the associated polynomial function is injective, i.e., it maps distinct arguments to distinct values [37,38]. A related graphical technique involves analysis of the CRN species-reaction (SR) graph [39,40] (see Additional file 1 for a description of the SR graph and its associated multistationarity theorem). There is also a suite of computational methods utilizing the so-called deficiency theorems for CRNs that are implemented in the publicly-available Chemical Reaction Network Toolbox [41]. These deficiency theorems give conditions for the existence, multiplicity, and stability of CRN steady-states that may often be applied in a parameter-free context; for example, the deficiency zero theorem states that if a CRN has certain topological properties, then within each *compatibility class *(an invariant manifold in species concentration space in which solutions are bound and determined by initial conditions), there is exactly one steady-state with strictly positive concentrations, and this steady-state is locally asympotically stable [42]. Interested readers may refer to refs. [42-48] for details on the full range of theorems implemented in the CRNT.

This section has briefly introduced five tools that can be used to determine if a given regulatory network has the capacity for multiple equilibria: the Thomas conjecture and Kauffman's multistationarity conditions based on analysis of the network's interaction graph (denoted below as the IG-T and IG-K methods, respectively), a multistationarity theorem based on the structure of the CRN SR graph (denoted by SRG), a computational method that can establish an injective property for the CRN's polynomial function (denoted by INJ), and CRN theory as implemented by the Chemical Reaction Network Toolbox (denoted by CRNT). Below we use these tools to analyze several small networks consisting of one gene and one gene product (the one-component subnetworks), in order to establish both the effectiveness of the various methods and the subnetworks' capacity for multistability. We then investigate a large set of more complex two-component subnetworks, including the well-studied 'double negative' feedback system. These small networks are studied without regard for their frequency of occurrence in the larger regulatory machinery of real biological systems, that is, we do not restrict ourselves to overrepresented network motifs. Lastly, we revisit the epidermal differentiation system in *Arabidopsis *as a real-world example of the applicability of these techniques.

## Results and Discussion

### Bistability in two positive autoregulatory subnetworks: a comparison of methods

**Figure 3:**
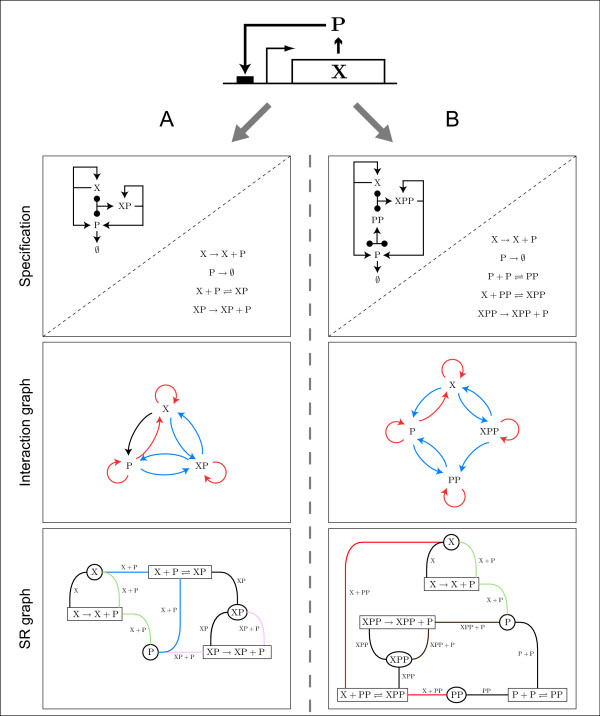
**Two specifications of a simple positive autoregulatory subnetwork**. A positive autoregulatory motif consists of a gene X that is transcribed and translated into transcription factor P, which in turn positively regulates the transcription of X by binding to a single *cis*-regulatory element in its promoter as either (A) a monomer or (B) a dimer. For (A), the motif can be specified as a CRN that includes basal production of P from X (X → X + P), degradation of P (P → ∅), binding and dissociation of P from the promoter of X (X + P ⇌ XP), and production of P by the transcription factor-gene complex XP (XP → XP + P). For (B), there is also basal production of P from X and degradation of P, however two transcription factors now associate to form a homodimer (P + P ⇌ PP), and it is this PP dimer that binds to the promoter of X (X + PP ⇌ XPP) and produces P via the dimer-gene complex XPP (XPP → XPP + P). In both cases the motif can also be visualized as wiring diagrams. The corresponding interaction graphs contain positive interactions (blue), negative interactions (red), and a variable interaction that can be either positive or negative, depending on the concentration of the transcription factor P (black). In the SR graphs for these CRNs, species are indicated by circles and reactions by rectangles, with the participation of a species in a reaction denoted by undirected edges labelled with the participating complex. Colored edges indicate a c-pair.

Consider a small regulatory network consisting of a single TF gene X that is transcribed and translated into protein P, which in turn positively regulates the production of X by binding to one or more independent *cis*-regulatory elements in its promoter. Such a module, commonly known as a positive autoregulatory motif and shown schematically in Fig. 3, is abundant in the transcriptional regulatory networks of eukaryotes (e.g., [1]). Simple representations such as that shown in Fig. 3 hide a significant amount of detail; for example, experimental and computational methods commonly used to establish network architecture usually fail to determine whether TFs bind to DNA as monomers, dimers, or as part of higher order structures. However, in the case of positive autoregulation, the actual form in which P binds to the promoter of X—as a monomer (Fig. 3A) or as a dimer (Fig. 3B)—has significant implications for the possibility of multistable behavior.

In Fig. 3A, the motif is specified as a CRN that includes basal production of P (X → X + P), degradation of P (P → ∅), reversible binding of P to the promoter of X (X + P ⇌ XP), and production of P by the TF-gene complex XP (XP → XP + P). In Fig. 3B there is also basal production of P from X and degradation of P, however two TFs now associate to form a homodimer (P + P ⇌ PP), and it is this PP dimer that binds to the promoter of X (X + PP ⇌ XPP) and produces P via the dimer-gene complex XPP (XPP → XPP + P). From these sets of elementary reactions, which following ref. [49] can also be visualized as wiring diagrams (see second row of Fig. 3), one can write an unambiguous system of ODEs that take the form of a CRN (Eq. 3). Although it has been shown that transcription and elements of post-transcriptional control combine to regulate the protein production rate, and that stochasticity exists at all levels of regulation [50], for simplicity we model protein production as a single deterministic process (cf. [51]). We address the role of translation and mRNA degradation in determining the capacity for multiple stable states in the next section.

For both Fig. 3A (the monomer model) and Fig. 3B (the dimer model), the existence of positive circuits in the interaction graph (e.g., X-XP-X and P-XP-P in Fig. 3A and X-XPP-X and P-PP-P in Fig. 3B) means that the IG-T theorem does not preclude multiple equilibria. Neither does the IG-K theorem preclude multiple equilibria; in Fig. 3A, there is a variable nucleus that includes the variable circuit X-P-X, and in Fig. 3B, there are two nuclei of opposite signs (the positive nuclei composed of circuits P-PP-P, X-X, and XPP-XPP, and the negative nuclei composed of circuits P-PP-P and X-XPP-X). (Eliminating one equation using the conserved quantities [X] + [XP] (Fig. 3A) and [X] + [XPP] (Fig. 3B) does not change the results of the IG-T and IG-K analysis; see Additional file 1.) The SRG method is also unable to rule out bistability in these motifs, in Fig. 3A due to the splitting of the X + P complex pair (or c-pair) associated with reaction X → X + P by two even-cycles (one composed of X and X → X + P, and the second composed of X, X → X + P, P, and X + P → XP), and in Fig. 3B because the cycle containing P and P + P ⇌ PP is not a 1-cycle or an odd-cycle. In addition, an injective property cannot be established for either model's polynomial function.

**Figure 4:**
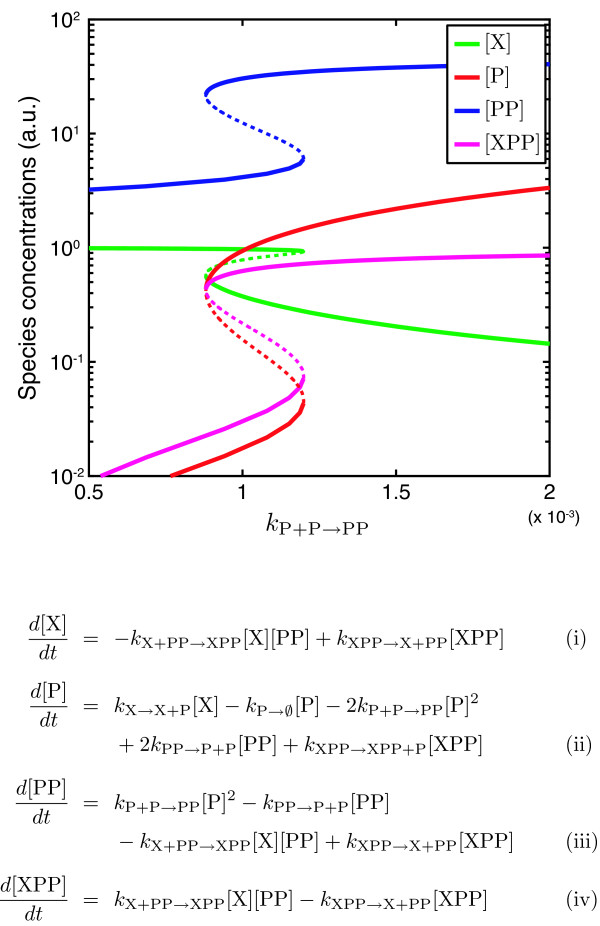
**Bistable autoregulatory motif**. The autoregulatory motif shown in Fig. 3B admits two stable equilibria, as shown in this bifurcation diagram. Stable steady states are denoted with solid lines, and unstable steady states are denoted with dashed lines. Equilibrium concentrations for X, P, PP, and XPP are plotted as a function of the association rate constant for dimer formation. The mass-action ODEs for this motif are also given. The other parameters obtained from the CRNT are: *k*_X→X+P _= 2.81, *k*_P→∅ _= 1, *k*_PP→P+P _= 0.98, *k*_X+PP→XPP _= 2.76, *k*_XPP→X+PP _= 1.55, and *k*_XPP→XPP+P _= 46.9. The unimolecular rate constants and dissociation rate constants are in units of time^-1^, while the bimolecular association rate constants are in units of conc^-1^time^-1^.

Unlike the IG-T, IG-K, SRG, and INJ methods, the CRNT determined that the monomer model cannot admit multiple equilibria for any choice of parameters. Conversely, the CRNT found that the dimer model does admit multiple equilibria and generated a set of parameters for which the motif is bistable. The dimer model mass-action ODEs are shown in Fig. 4, along with a bifurcation diagram of equilibrium species concentrations as a function of the dimer association rate constant (see caption for parameters). While it has been previously demonstrated through analysis of a positive autoregulatory motif model with explicit mRNA concentration that dimerization is necessary for bistability [52], it is significant that modeling transcription and translation as a single operation leads to similar results.

The bistability of the dimer version of the autoregulatory motif, confirmed using CRNT, is consistent with the inability of the IG-T, IG-K, SRG, and INJ methods to rule it out. However, these methods are also unable to rule out bistability in even the simple case of positive autoregulation by a protein monomer, suggesting that they may be of limited use in the analysis of more complex systems.

### Analysis of twelve one-component subnetworks

We extended the preceding analysis to ten additional one-component subnetworks constructed from various subsets of the elementary reactions listed in Table 1 (see Additional file 1 for the specification rules). Table 2 shows that the IG-T, IG-K, and SRG methods give little information about the capacity for multistability in these twelve simple subnetworks. Indeed, all that can be determined using these methods is the trivial result that multiple equilibria requires the reversible binding of TFs to the promoters of the component genes; constitutive expression alone is not sufficient. The IG-T and IG-K methods can only rule out mutistability for subnetwork *ab *(a minimal network containing only basal production and degradation of P). The SRG method only rules out multistability in subnetworks *ab *and *abe *since the SR graphs contain only a single cycle and thus there are no split c-pairs (see Additional file 1: Fig. S4).

**Table 1:** Construction of one-component regulatory subnetworks

**Reaction label**	**Reaction**
*a*	X → X + P
*b*	P → ∅
*c*	X + P ⇌ XP
*d*	XP → XP + P
*e*	P + P ⇌ PP
*f*	X + PP ⇌ XPP
*g*	XPP → XPP + P

Seven reactions that are combined to generate twelve subnetworks with one gene and one gene product.

**Table 2:** Survey of one-component regulatory subnetworks

**Subnetwork**	**Dimer formation**	**Monomer binding**	**Dimer binding**	**Multiple equilibria ruled out?**				
				
				**IG-T**	**IG-K**	**SRG**	**INJ**	**CRNT**
*ab*	*no*	*no*	*no*	*yes*	*yes*	*yes*	*yes*	*yes*
*abcd*	*no*	*yes*^+^	*no*	*no*	*no*	*no*	*no*	*yes*
*abc*	*no*	*yes*^-^	*no*	*no*	*no*	*no*	*yes*	*yes*
*abe*	*yes*	*no*	*no*	*no*	*no*	*yes*	*yes*	*yes*
*abefg*	*yes*	*no*	*yes*^+^	*no*	*no*	*no*	*no*	*no*
*abef*	*yes*	*no*	*yes*^-^	*no*	*no*	*no*	*yes*	*yes*
*abcde*	*yes*	*yes*^+^	*no*	*no*	*no*	*no*	*no*	*yes*
*abcdefg*	*yes*	*yes*^+^	*yes*^+^	*no*	*no*	*no*	*no*	*no*
*abcdef*	*yes*	*yes*^+^	*yes*^-^	*no*	*no*	*no*	*no*	*yes*
*abce*	*yes*	*yes*^-^	*no*	*no*	*no*	*no*	*yes*	*yes*
*abcef*	*yes*	*yes*^-^	*yes*^-^	*no*	*no*	*no*	*yes*	*yes*
*abcefg*	*yes*	*yes*^-^	*yes*^+^	*no*	*no*	*no*	*no*	*no*

Analysis of twelve gene regulatory subnetworks that consist of one gene and one gene product. The letters that make up the *Subnetwork *names refer to the constituent reactions listed in Table 1. The entries *yes*^+/- ^indicate productive/unproductive binding to the gene promoter. The five analytical tools IG-T, IG-K, SRG, INJ, and CRNT are described in the text. In the three cases where multiple equilibria were not ruled out (subnetworks *abefg*, *abcdefg*, and *abcefg*), the CRNT provided example sets of rate constants leading to bistability.

In contrast to these graphical methods, the INJ ruled out multistability in six of twelve cases, and the CRNT ruled out multistability in nine of twelve cases. Furthermore, for the three subnetworks where multiple equilibria were not ruled out by the CRNT (*abefg*, *abcdefg*, and *abcefg*), parameter sets leading to bistability were provided. In these two methods we begin to see the strength of the full chemical reaction network theory: with the assumption of mass-action kinetics, the underlying structure of CRNs limits the nonlinearities that appear in the full ODE model [48]. The bifurcation diagram for subnetwork *abefg *is shown in Fig. 4. Similar results for subnetworks *abcdefg *and *abcefg *are shown in Additional file 1, Fig. S5.

As previously mentioned, the twelve subnetworks presented in Table 2 were constructed under the assumption that, for the purpose of analyzing subnetwork equilibria, transcription and translation may be combined into a single operation. We repeated our analysis using one-component subnetworks augmented by elementary reactions that make these processes explicit. The elementary reactions X → X + P, XP → XP + P, XPP → XPP + P were replaced by reactions representing transcription (X → X + R, XP → XP + R, XPP → XPP + R), translation (R → R + P), and mRNA degradation (R → ∅). Application of the CRNT to these augmented one-component systems led to no change in the results of Table 2, and thus we model protein production as a single one-step process for the remainder of this paper.

### Survey of two-component regulatory subnetworks

Consideration of small networks consisting of two genes (X_1 _and X_2_) and two gene products (P_1 _and P_2_) is a natural extension of the analysis presented above. With a second component included, the number of relevant subnetworks increases to 40,680 (Additional file 1, Table S1 lists the constituent reactions). The subnetworks may be grouped by their number of dimer/promoter association reactions; every two-component subnetwork has between zero and six such reactions (two homodimers and one heterodimer which can each bind to either promoter). For bistability analysis, we applied the INJ method in an automated fashion using Matlab scripts provided by G. Craciun [37]. No attempt was made to apply the IG-T, IG-K, and SRG methods that were found to be uninformative with respect to subnetworks with one-component (Table 2). Approximately 15,000 subnetworks were randomly selected for study, sampled in a manner that ensured that subnetworks with zero to six dimer-binding reactions were represented in the same proportion as in the complete set of subnetworks.

Surprisingly, we found that the percentage of two-component subnetworks for which bistability could be ruled out using the INJ method drops to only 6.71%, with a 95% bootstrap confidence interval [6.31%, 7.11%], down from 50% in the one-component systems. As shown in Table 3, partitioning the results according to the number of dimer-binding reactions reveals the importance of TF dimerization and feedback to multistability: the group with no dimers binding has the highest percentage of subnetworks that cannot be bistable (26.2% with confidence interval [19.0%, 34.4%]), and as the number of dimer-binding reactions increases, the percentage of subnetworks definitively without multiple equilibria decreases. Multistability could not be ruled out for any of the sampled two-component subnetworks that contain six dimer-promoter binding reactions.

**Table 3:** Bootstrap analysis results for two-component regulatory subnetworks

**# dimers binding**	**% of total models**	**% with multiple equilibria ruled out**
0	0.8	26.2 [19.0, 34.4]
1	4.8	19.8 [17.1, 22.9]
2	14.5	12.6 [11.3, 14.1]
3	25.5	8.9 [8.1, 9.9]
4	28.8	4.1 [3.5, 4.7]
5	19.1	1.4 [1.0, 1.9]
6	6.5	0.0

The percentage of two-component subnetworks for which multiple equilibria can be ruled out, based on INJ analysis of ~15,000 randomly selected subnetworks (see text). Results are grouped according to the number of dimer-binding reactions in each subnetwork. Brackets indicate bootstrap 95% confidence intervals calculated by sampling with replacement 10,000 times. Overall, the capacity for multiple equilibria is ruled out by the INJ method for 6.71% [6.31%, 7.11%] of sampled subnetworks.

In the preceding analysis, we did not distinguish between productive and unproductive TF binding reactions. We define a productive reaction as one in which expression of the corresponding gene is activated. Similarly, an unproductive reaction is one in which gene expression is repressed. When the analysis is applied to subsets of systems lacking either productive or unproductive TF binding, the percentage of sampled subnetworks for which INJ could note rule out bistability increases as the number of binding reactions increases (see Additional file 1, Tables S3 and S4). Note that the INJ method is more successful at ruling out bistability for subnetworks lacking productive TF binding than it is for subnetworks lacking unproductive TF binding, perhaps because bistable networks are enriched in productive TF-promoter interactions.

The CRNT was found to be the most effective method for establishing and ruling out bistability in the one-component subnetworks. However, the current release of the CRNT cannot be automated, and is thus difficult to apply to a large set of two-component systems. For this reason we randomly selected 25 two-component subnetworks and applied the INJ and CRNT methods to compare their effectiveness. The CRNT found 19 subnetworks that do not support multiple equilibria (76% with confidence interval [52%, 88%]), and it generated parameter sets that lead to bistability for 5 of the remaining 6 subnetworks (20% of total with confidence interval [4%, 36%]). This is quite similar to the fraction of one-component subnetworks that were found by the CRNT to be bistable (25%) and well within the confidence interval. Conversely, the INJ method was only able to rule out bistability for 3 of the 19 subnetworks ruled out by the CRNT (Additional file 1, Table S2). Although derived from a small sample, taken together these results suggest that the INJ method is debilitated by network size to a larger degree than the CRNT method.

**Figure 5:**
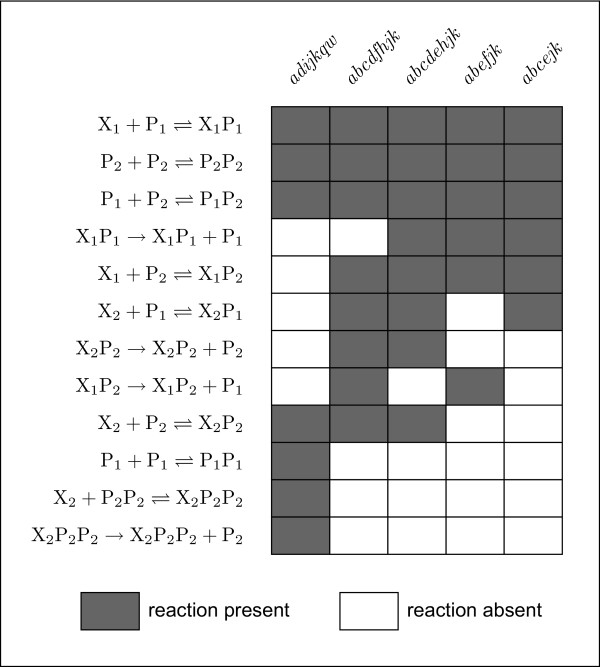
**Bistable two-component subnetwork reactions**. The 5 two-component subnetworks that CRNT established can support bistability (*abcdfhjk*, *abcdehjk*, *abcejk*, *adijkqw*, and *abefjk*) are compared. All 5 subnetworks contain binding of the X_1 _gene by P_1 _and dimerization of P_2 _by both P_1 _and P_2_. (The basal production and degradation reactions are also common to all but are not shown explicitly.) A number of other reactions are common to various subnetworks.

The five two-component subnetworks that CRNT established can support bistability (*abcdfhjk*, *abcdehjk*, *abcejk*, *adijkqw*, and *abefjk*) are compared in Fig. 5. It can be seen that all five subnetworks contain binding of the X_1 _gene by P_1 _and dimerization of P_2 _by both P_1 _and P_2_. The basal production and degradation reactions are also common to all but are not shown for clarity. A number of other reactions are common to various subnetworks. It would appear that one common feature between all of these bistable systems is sequestration of the productive TF(s) into inactive complexes. In subnetwork *abcejk *for example, P_1 _binds productively to X_1_, but it also complexes with itself, with P_2_, and it binds unproductively to X_2_. P_2 _also binds unproductively to X_1_, competing with P_1_. Interestingly, only *adijkqw *includes a TF dimer-promoter binding reaction, shown to be essential for bistability in the one-component systems. Though not comprehensive, this analysis does suggest the existence of a large number of different network architectures capable of supporting bistability.

### The canonical reciprocal repression subnetwork

**Figure 6:**
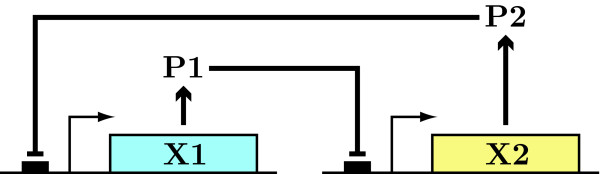
**Two-component reciprocal repression**. A reciprocal repression subnetwork consists of pair of genes (X_1 _and X_2_) in which each gene's product blocks transcription of the other gene, either as a monomer (X_*i *_+ P_*j *_⇌ X_*j*_P_*j *_for *i *≠ *j*) or a dimer (P_*i *_+ P_*I *_⇌ P_*i *_P_*i *_and X_*i *_+ P_*j *_P_*j *_⇌ X_*i *_P_*j *_P_*j *_for *i *≠ *j*). Basal protein production (X_*i *_→ X_*i *_+ P_*i*_) and protein degradation (P_*i *_→ ∅) are also included in specification of the subnetwork.

We turn our attention to the 'double-negative' feedback system (also known as a reciprocal repression system) first described by Monod and Jacob [53]: a small network consisting of pair of genes (X_1 _and X_2_) in which each gene's product inhibits transcription of the other gene (Fig. 6). It has been shown both theoretically [54] and experimentally [55] that cooperativity in TF binding is required for this subnetwork to exhibit bistable behavior. An analysis of CRN specifications of this small network is thus an excellent test of the techniques discussed herein. We constructed two representatives of this subnetwork: one with regulation via TF monomers (X_*i *_+ P_*j *_⇌ X_*i *_P_*j *_for *i *≠ *j*) and the other with TF dimerization and regulation via TF dimer-promoter interactions (P_*i *_+ P_*i *_⇌ P_*i*_P_*i *_and X_*i *_+ P_*j *_P_*j *_⇌ X_*i*_P_*j *_P_*j *_for *i *≠ *j*). Both models also contained basal production (X_*i *_→ X_*i *_+ P_*i*_) and degradation (P_*i *_→ ∅) of TFs. CRNT analysis confirmed that the subnetwork containing TF dimerization exhibits bistability for a suitable set of parameters, while the subnetwork with double-negative feedback via TF monomers does not have this capacity.

### Revisiting the trichome differentiation subnetwork with the CRNT

As described above and shown in Fig. 1, the trichome differentiation subnetwork consists of regulators belonging to the R2R3-MYB (e.g., GL1) and bHLH (e.g., GL3) classes that directly regulate GL2 and CPC among several other targets [3]. As there is no experimental evidence for the direct positive feedback that has been posited in current models of trichome initiation, the question of whether the system can support bistability without it is one of great interest.

We specify the core trichome differentiation subnetwork as a CRN that contains reaction equations for basal expression and degradation of CPC, formation of the GL3-GL1 and GL3-CPC complexes, and binding of a single GL3-GL1 complex to the promoter of CPC to positively regulate its production. The constituent equations are given in Table 4 (Model 1). On application of the CRNT, we found that this subnetwork specification cannot support bistability. However, we have shown that details of TF-promoter interactions can be a critical determinant for a regulatory network to exhibit multiple stable equilibria. We thus constructed three additional CRN specifications of the trichome differentiation subnetwork that allow for the possibility of dimerization of the GL3-GL1 active complex. As with the monomer-binding model described above, each of these models contains basal expression and degradation of CPC, and GL3-GL1 and GL3-CPC complex formation. In Model 2, GL3-GL1 can only bind to the promoter of CPC and activate its transcription as a complex dimer (heterotetramer) that forms prior to TF-promoter interaction. Model 3 contains sequential binding of two GL3-GL1 complexes to the separate sites in the CPC promoter, with CPC protein being produced only when both complexes are bound. Model 4 is a hybrid of Model 1 and Model 2, where the GL3-GL1 can bind to the CPC promoter as a monomer or a dimer, and both can activate transcription of CPC. The constituent equations for these models are also given in Table 4. In contrast to the monomer-binding case, we found Models 2-4 are all capable of exhibiting bistability, even in the absence of direct positive autoregulation.

**Table 4:** CRNs consistent with the core trichome differentiation network

**Reactions**	**Model 1**	**Model 2**	**Model 3**	**Model 4**
X → X + CPC	✔	✔	✔	✔
CPC → ∅	✔	✔	✔	✔
GL3 + GL1 ⇌ GL3-GL1	✔	✔	✔	✔
GL3 + CPC ⇌ GL3-CPC	✔	✔	✔	✔
GL3-GL1 + GL3-GL1 ⇌ GL3-GL1-GL3-GL1		✔		✔
GL3-GL1 + X ⇌ GL3-GL1-X	✔		✔	✔
GL3-GL1 + GL3-GL1-X ⇌ GL3-GL1-GL3-GL1-X			✔	
GL3-GL1-X → GL3-GL1-X + CPC	✔			✔
GL3-GL1-GL3-GL1 + X ⇌ GL3-GL1-GL3-GL1-X		✔		✔
GL3-GL1-GL3-GL1-X → GL3-GL1-GL3-GL1-X + CPC		✔	✔	✔

Result of CRNT analysis:	**not bistable**	**bistable**	**bistable**	**bistable**

Constituent reactions for four specifications of the trichome differentiation network (Fig. 1).

## Conclusion

We have used a modeling framework in which small gene regulatory networks are specified as chemical reaction networks (CRNs), with reactions describing transcription factor (TF) production, degradation, dimerization, and association of both monomer and dimer TFs with promoters in a productive or unproductive fashion. We used these methods to survey twelve subnetworks consisting of one TF gene and gene product (the one-component subnetworks) and found that bistability is only exhibited by those that include positive feedback with cooperative TF binding (Table 2). This is not unexpected, as direct positive feedback and non-linearity are often prerequisites for bistability in simple systems [15,20]. When the analysis tools were applied to a large random sample of two-component subnetworks (from a total of 40,680 consistent with our CRN specification rules; see Additional file 1), we found that the presence of cooperative TF binding makes it more difficult to rule out bistability (Table 3), although it is ruled out more often in subnetworks lacking direct positive feedback than it is for those lacking direct negative feedback (Additional file 1, Tables S3 and S4). Interestingly, we found several examples of bistable two-component subnetworks that do not include promotor-TF dimer interactions at all (Fig. 5 and Additional file 1, Table S2), consistent with prior work demonstrating that sequestration and titration of active TFs into inactive complexes can give rise to non-linearity and bistable behavior [51,56].

Our application of the various equilibria analysis tools suggests that the interaction graph-based techniques (IG-T and IG-K) that are generally applicable to deterministic ODE systems are rarely informative with respect to small gene regulatory networks. We also found that the deficiency theorems implemented in the Chemical Reaction Network Toolbox (CRNT) are generally more informative than computational (INJ) and graphical (SRG) methods that attempt to rule out multiple equilibria by establishing injectivity of the reaction network polynomial function. We emphasize that the success of the CRNT is partly due to the special form of chemical reaction networks that assume kinetics of the mass-action form. However, this assumption would appear to be a valid one under many working conditions. That the CRNT accurately determined the stability properties of the well-studied positive autoregulatory and reciprocal repression systems (Fig. 3 and Fig. 6, respectively), and that one-component systems found by CRNT to be bistable were confirmed as such with bifurcation diagrams (Fig. 4 and Additional file 1, Fig. S5), is further validation of the CRNT's usefulness.

It is important to note that the analytical methods described herein are not the only ones available for probing the stability properties of a dynamical system. One additional method involves evaluation of the Jacobian matrix (Eq. 2), because the existence of a saddle-node bifurcation leading to stable and unstable equilibria requires the Jacobian to be singular at the critical point *c*_*ss *_(i.e., det{*J*(*c*_*ss*_)} = 0) [57]. We do not use continuation methods to directly rule out saddle-node bifurcations in this work, as our attempts at such analysis have not yielded results, and to our knowledge it would be difficult to explore large numbers of regulatory networks in this fashion. Furthermore, deterministic ODEs are only one of several mathematical structures used to model gene regulatory networks; other possibilities include boolean networks, Petri nets, Markov chains, stochastic ODEs, and so on. Still, the parameter-free approach to modeling that we have emphasized here is an attractive one for at least two reasons: (1) there are often multiple assumptions made (such as the validity of Michaelis-Menten or Hill-type expressions) when complex network models are constructed as a generic system of ODEs, and these assumptions may result in inconsistencies [49], and (2) as we have shown, CRN theory facilitates analysis that is usually more fruitful than techniques available for generic ODEs.

The current state of CRN-based modeling and analysis does not include stochastic effects, despite the fact that there is both experimental and theoretical evidence that low concentrations of TFs or binding sites can lead to fluctuations (i.e, noise) that may sometimes be an essential aspect of network function [50,58-60]. The CRN approach thus represents a first step towards generating mixed models of biological systems that incorporate stochastic effects. Future work could extend CRN-based techniques to include stochastic dynamics consistent with the elementary processes that compose gene regulatory networks [61]. We further anticipate that new releases of the CRNT software package will also be amenable to automation and will allow for CRNs of unlimited size.

Although the modeling formalism used here is a simple one, it is not clear that an increase in complexity would lead to different conclusions; for example, we have already demonstrated that excluding mRNA does not influence the one-component subnetwork results. Two other simplifications made find significant experimental support: (i) that TF dimers are stable to proteolytic degradation (cf. [51,56]), a reasonable assumption given that protein dimerization typically provides protection against proteolysis by enhancing thermal stability or through blocking access to monomer degradation tags [52], and (ii) the exclusion of higher-order oligomeric TF complexes (such as trimers or tetramers), an assumption appropriate for many eukaryotic TF gene families where dimerization is sufficient for functionality [62]. On the other hand, TF activation can occur by posttranslational modification (e.g., phosphorylation [51]), a situation analogous to cell signaling pathways in which dual phosphorylation/dephosphorylation cycles confer bistability [63]. While it is possible to extend the modeling formalism illustrated here in these and other directions, the additional combinatorial complexity would make surveys of multi-component networks computationally challenging.

Applying the CRNT to the real-world example of *Arabidopsis *epidermal cell differentiation, we have shown that gene regulatory subnetworks consistent with established components and interactions may have the capacity for bistability. This capacity of course depends on the specific details of the network architecture. In particular, we found that dimerization of the active GL3-GL1 complex and cooperative sequential binding of GL3-GL1 to the CPC promoter are each independently sufficient to generate a bistable subnetwork, even in the absence of direct positive autoregulation. Both of these alternatives have some experimental support. Sequential binding of GL3-GL1 to the CPC promoter is plausible, as multiple TF binding sites in gene promoters are a general characteristic of eukaryotic gene expression [64]. And while dimerization of GL3-GL1 has not been experimentally demonstrated, GL3 can dimerize [65] either through the conserved bHLH domain, as is the case for other bHLH factors [66], or through the conserved C-terminal ACT domain [67]. Bistability may also be achieved if the GL3-GL1 complex is not stable against proteolysis (not shown).

If it can be determined that GL3-GL1 does not in fact dimerize or bind to two separate binding sites in the CPC promoter, and that it is in fact a stable complex, then the current view of the core trichome differentiation network (Fig. 1) must be incomplete. In that case, there are several intriguing possibilities. It may be that one or more of the many additional trichome-related genes that have been recently identified [3] are as central to trichome differentiation as GL3, GL1, and CPC. It is possible that epidermal cell differentiation in *Arabidopsis *is not in fact subserved by a bistable gene regulatory network. It may also be that some unknown extrinsic mechanism plays a role in *Arabidopsis *epidermal cell fate determination. A mechanism of this kind may render a bistable network irreversible by causing one equilibrium state to be inaccessible [21], or it may fix a transient activated state before the system can return to a basal equilibrium [68]. There is some evidence for these two latter possibilities, as a differentiated trichome cell is unlikely to revert back to an undifferentiated state. In particular, a recent study has shown that the induction of GL3 function for 4 hrs is sufficient to trigger trichome initiation, with development continuing even after GL3 function is completely removed [29]. Clearly there are a number of possibilities that we have only begun to investigate. Future experiments will be required to distinguish between them and may yet provide insights into this interesting developmental model system.

## Methods

### Analyzing bistability with the CRNT

The Chemical Reaction Network Toolbox (CRNT) is a stand-alone DOS program that uses CRN theory [42,44-47] to to determine if a given CRN has the capacity for multiple equilibria; if so, it will often provide example parameter values (see ref. [41]). In Additional file 1 we detail rules designed to specify families of a gene regulatory networks of interest (e.g., the one-component subnetworks shown in Tables 1 and 2, each of which is a CRN). To analyze a subnetwork using CRNT, we list the species, complexes (including the null complex ∅), and reactions, and enter them manually into the CRNT Network Analyst. With the currently available implementation of the CRNT we were restricted to the analysis of subnetworks containing twenty complexes or less. For most subnetworks discussed here, the CRNT Advanced Deficiency Theory option is needed following the initial analysis.

### Generation of one- and two-component regulatory subnetworks

One- and two-component subnetworks were generated in the Matlab programming environment (The MathWorks, Inc.). One-component networks are distinguished by whether TF monomer can bind the promoter and, if monomer binding does occur, whether the monomer-promotor complex results in transcription; thus there are three options with respect to monomer binding (*no*, *yes*^-^, *yes*^+^; see Table 2). One-component networks are further distinguished by whether or not dimers can form (*no*, *yes*) and, if so, whether the dimer-promoter complex is productive (*no*, *yes*^+^, *yes*^-^). Because this latter question is only relevant when dimer can form, there are 3 + 3.3 = 12 distinct one-component networks.

The generation of two-component networks involves enumerating all possibilities with regard to homodimer and heterodimer formation, being careful to avoid double counting of networks that are equivalent via symmetry, i.e., when all species (X_1_, X_2_, P_1_, P_2_, etc.) are relabeled by exchanging the subscripts 1 and 2. The heterodimers P_1_P_2 _and P_2_P_1 _were assumed to be equivalent. These distinct possibilities for homodimer and heterodimer formation were subsequently instantiated multiple times to account for each possible pattern of monomer and dimer binding, resulting in 40,680 two-component networks.

## Authors' contributions

DSG and GS performed all the computational and mathematical analyses. EG and GS were involved in the initial development of the concept. All authors contributed to the manuscript writing and have read and approved the final manuscript.

## Supplementary Material

Additional file 1 **The capacity for multistability in small gene regulatory networks: Supplementary Materials**. Additional notes on the mathematical tools used and the role of positive and negative feedback in bistability. Also included are the SR graphs for the one component networks. Click here for file
