# Supplementary material for: The capacity for multistability in small gene regulatory networks
Source: BMC Syst Biol. 2009 Sep 21;3:96. doi: 10.1186/1752-0509-3-96 (PMC2759935; doi:10.1186/1752-0509-3-96)
Supplement: Additional file 1 — The capacity for multistability in small gene regulatory networks: Supplementary Materials. Additional notes on the mathematical tools used and the role of positive and negative feedback in bistability. Also included are the SR graphs for the one component networks. [file 1752-0509-3-96-S1.PDF]

The capacity for multistability in small gene regulatory networks

Dan Siegal-Gaskins[1,2], Erich Grotewold[1,2]and Gregory D. Smith*[1,3]

[1]Mathematical Bioscience Institute, The Ohio State University, Columbus, OH 43210 USA
[2]Department of Plant Cellular and Molecular Biology and Plant Biotechnology Center, The Ohio State University, Columbus, OH 43210 USA
[3]Department of Applied Science, The College of William and Mary, Williamsburg, VA 23187 USA

Email: Dan Siegal-Gaskins - dsg@mbi.ohio-state.edu; Erich Grotewold - grotewold.1@osu.edu; Gregory D. Smith* - greg@as.wm.edu;

*Corresponding author

Supplementary Materials

Additional comments on the injectivity (INJ) method as a tool for ruling out multistability

The INJ method used in this paper takes advantage of the conditions sufficient for unique equilibria originally described in refs. [1, 2]. These conditions were developed in the context of modeling an isothermal homogenous continuous flow stirred tank reactor (CFSTR), essentially an enclosed liquid phase volume in which chemical reactions occur, each species is supplied at a constant volumetric flow rate, and contents are removed at the same volumetric flow rate. While a feed stream and effluent stream are not part of our gene regulatory network modeling framework, the INJ method is still applicable: it has been shown that if a system with constant-rate-production and degradation cannot have multiple equilibria, then any subsystem without some (or all) of those processes also cannot have multiple equilibria, so long as the subsystem formed by removing all species that are not entrapped has the *normality* property (see Ref. [3]). All the one- and two-component networks described in this work include the reaction $\mathrm{P}_i \rightarrow \emptyset$, which can be interpreted as an efflux. The 'entrapped-species projections' of these networks include only association of dimers to promoters ($\mathrm{X}_i + \mathrm{P}_i\mathrm{P}_j \rightleftharpoons \mathrm{X}_i\mathrm{P}_i\mathrm{P}_j$, and these projections are all reversible, which is a sufficient condition for normality [3].

The Species-Reaction (SR) graph

The SR graph of a CRN is a construct that can sometimes be used to establish that the associated system of differential equations cannot admit multiple positive equilibria, regardless of the parameter values [2, 4].

Each species in the network is represented in the graph by a species node. Similarly, each reaction or reversible reaction pair is represented by a reaction node. The combinations of species at the heads or tails of the reaction arrows are referred to as complexes. Note that a complex can be a single species (as in the left hand side of the equation $\mathrm{X} \rightarrow \mathrm{X} + \mathrm{P}$) or a combination of species (as in the right hand side of the same equation). Undirected edges are drawn between species and reaction nodes if the species appears in a complex participating in the reaction. Edges are labelled with the name of the participating complex, and carry the stoichiometric coefficient of the species in the complex. If a species occurs in both sides of the reaction, two edges are drawn (e.g., in Fig. 3A two edges connect species XP to reaction $\mathrm{XP} \rightarrow \mathrm{XP} + \mathrm{P}$, one labelled with complex XP and the other with complex XP + P). The SR graphs for subnetworks *abcd* and *abefg* are shown in Fig. 3, and the SR graphs for all the one-component subnetworks described in the text are shown in Supplementary Figures.

If two edges that meet at a reaction node have the same complex label, this pair is referred to as a c-pair (for complex pair). Also, SR graphs may contain cycles, closed series of edges in which no edge or node is traversed more than once. Cycles may be odd or even depending on the number of contained c-pairs. Furthermore, if all the stoichiometric coefficients along a cycle are 1, then that cycle is said to be a 1-cycle. Lastly, if an SR graph contains at least two cycles, with the union of the two cycles containing a c-pair, and at least one of the cycles containing only one of the edges of the c-pair, then that c-pair is said to be split between the two cycles.

To analyze a network using the SRG method, we (manually) apply the theorem presented in [4]. The theorem states that multistationarity can be ruled out for a CRN when the corresponding SR graph has both of the following properties: (1) each cycle in the graph is a 1-cycle, an odd-cycle, or both, and (2) no complex-pair (c-pair) is split by two even-cycles.

Subnetwork size reduction using conserved quantities

In the small networks modeled here, the total concentration of each gene in free and bound forms is conserved. This allows the elimination of an ODE and leads to simplified interaction graphs. For example, the complete set of ODEs describing subnetwork *abcdefg* are

$$\frac{d[\mathrm{X}]}{dt} = -k_3[\mathrm{X}][\mathrm{P}] + k_4[\mathrm{XP}] - k_8[\mathrm{X}][\mathrm{PP}] + k_9[\mathrm{XPP}] \quad \text{(S1)}$$

$$\frac{d[\mathrm{P}]}{dt} = k_1[\mathrm{X}] - k_2[\mathrm{P}] - k_3[\mathrm{X}][\mathrm{P}] + k_4[\mathrm{XP}] + k_5[\mathrm{XP}] - 2k_6[\mathrm{P}]^2 + 2k_7[\mathrm{PP}] + k_{10}[\mathrm{XPP}] \quad \text{(S2)}$$

$$\frac{d[\mathrm{XP}]}{dt} = k_3[\mathrm{X}][\mathrm{P}] - k_4[\mathrm{XP}] \quad \text{(S3)}$$

$$\frac{d[\mathrm{PP}]}{dt} = k_6[\mathrm{P}]^2 - k_7[\mathrm{PP}] - k_8[\mathrm{X}][\mathrm{PP}] + k_9[\mathrm{XPP}] \quad \text{(S4)}$$

$$\frac{d[\mathrm{XPP}]}{dt} = k_8[\mathrm{X}][\mathrm{PP}] - k_9[\mathrm{XPP}]. \quad \text{(S5)}$$

However, we also have that $[\mathrm{X}] + [\mathrm{XP}] + [\mathrm{XPP}] = \mathrm{X}_{\mathrm{TOTAL}}$, so that either Eqs. S1, S3, or S5 can be eliminated. For example, eliminating Eq. S5 leads to the following set of ODEs

$$\frac{d[\mathrm{X}]}{dt} = -k_3[\mathrm{X}][\mathrm{P}] - k_8[\mathrm{X}][\mathrm{PP}] + k_9(\mathrm{X}_{\mathrm{TOTAL}} - [\mathrm{X}] - [\mathrm{XP}]) + k_4[\mathrm{XP}] \quad \text{(S6)}$$

$$\frac{d[\mathrm{P}]}{dt} = k_{10}(\mathrm{X}_{\mathrm{TOTAL}} - [\mathrm{X}] - [\mathrm{XP}]) + k_1[\mathrm{X}] - k_2[\mathrm{P}] - 2k_6[\mathrm{P}]^2 + 2k_7[\mathrm{PP}] - k_3[\mathrm{X}][\mathrm{P}] + (k_4 + k_5)[\mathrm{XP}] \quad \text{(S7)}$$

$$\frac{d[\mathrm{XP}]}{dt} = k_3[\mathrm{X}][\mathrm{P}] - k_4[\mathrm{XP}] \quad \text{(S8)}$$

$$\frac{d[\mathrm{PP}]}{dt} = k_6[\mathrm{P}]^2 - k_7[\mathrm{PP}] - k_8[\mathrm{X}][\mathrm{PP}] + k_9(\mathrm{X}_{\mathrm{TOTAL}} - [\mathrm{X}] - [\mathrm{XP}]). \quad \text{(S9)}$$

The elimination of Eq. S1 or Eq. S3 leads to similar sets of equations. When the interaction graph corresponding to these reduced equations are analyzed using the IG-T and IG-K methods, it is conceivable that they would be more informative than the interaction graph for the full equations. While such reduction simplifies application of the IG-T and IG-K methods, with the exception of subnetwork *abe* in which $d[\mathrm{X}]/dt = 0$, we found no example where new information became available upon re-analysis of a reduced system.

A set of rules for specifying regulatory networks as CRNs

The usual biological representation of a regulatory network (e.g., Fig. 3) is insufficient to unequivocally specify the reactions (and thus equations) that make up a chemical reaction network (CRN). To address this ambiguity, we restricted our surveys of one- and two-component subnetworks to CRNs that can be formulated in a manner consistent with the following rules:

1. The number N of gene/gene product pairs that participate in the network is specified (e.g., $N = 1$, Fig. 3A).

2. A non-zero basal production rate for each gene product is assumed ($\mathrm{X}_i \rightarrow \mathrm{X}_i + \mathrm{P}_i$ where $i = 1, 2, \cdots, N$). This basal production could be furnished by regulatory factors that are not explicitly modeled.

3. A non-zero degradation rate for each gene product is assumed ($\mathrm{P}_i \rightarrow \emptyset$).

4. The homo- and hetero-dimers that may be produced through association of gene products are specified. That is, we specify for which i and j (where $i \leq j$) the reaction $\mathrm{P}_i + \mathrm{P}_j \rightleftharpoons \mathrm{P}_i\mathrm{P}_j$ is included.

5. Homo- and hetero-dimers are assumed to be stable to degradation, being reduced in concentration only through dissociation of the components (see above).

6. We assume each gene contains a single binding site for monomers or for homo- or hetero-dimers of the gene products. This site can remain unoccupied or be occupied by either monomer or dimer. That is, we specify for which i and j the reaction $\mathrm{X}_i + \mathrm{P}_j \rightleftharpoons \mathrm{X}_i\mathrm{P}_j$ is included (monomer binding). And we specify for which i, j, and k (where $j \leq k$) the reaction $\mathrm{X}_i + \mathrm{P}_j\mathrm{P}_k \rightleftharpoons \mathrm{X}_i\mathrm{P}_j\mathrm{P}_k$ is included (dimer binding).

7. We specify whether the interactions of all the transcription factor species (monomer and dimer) with the binding site of each gene are productive or unproductive. That is, we specify for which i and j the reaction $\mathrm{X}_i\mathrm{P}_j \rightarrow \mathrm{X}_i\mathrm{P}_j + \mathrm{P}_i$ is included (productive monomer binding). And we specify for which i, j, and k (where $j \leq k$) the reaction $\mathrm{X}_i\mathrm{P}_j\mathrm{P}_k \rightarrow \mathrm{X}_i\mathrm{P}_j\mathrm{P}_k + \mathrm{P}_i$ is included (dimer binding).

These rules are meant to be illustrative and not prescriptive. The one-component (two-component) subnetworks considered in this paper satisfy the above criteria with the assumption one (two) gene/gene product pairs. These rules could be extended to include multiple regulatory sites per gene, and in this case one might for simplicity assume independent binding of homo- and hetero-dimers to these sites (i.e., without regard to the status of other binding sites associated with the same gene). The CRNs constructed to represent possible specifications of the core trichome differentiation network (Fig. 1) do not conform to these rules used to generate the one- and two-component systems considered here, for example, Model 2 and 4 of Table 4 include dimerization of the GL3-GL1 complex (a tetramer).

The role of positive and negative feedback in bistability

The vast majority of the two-component subnetworks contain some degree of direct positive/negative feedback, wherein binding of transcription factors to a promotor (as either monomers or dimers) increases/decreases the protein production rate. To determine the importance of positive feedback on the capacity for multistability, we analyzed a subset of systems that entirely lack productive TF binding (both monomers and dimers), reducing the number of sampled subnetworks from $\sim$15,000 to 542. Table S3 shows the results, which are similar to those from the previous analysis presented in Table 3. Note that an increase in dimer-binding reactions is associated with a decrease in the fraction of subnetworks for which multiple equilibria can be ruled out.

Table S4 shows similar results using the subset of small networks that lack unproductive TF binding, reducing the number of sampled subnetworks is reduced from $\sim$15,000 to 592. The INJ method is more successful at ruling out bistability for subnetworks that lack productive TF binding than subnetworks that lack unproductive TF binding.

References

1. Craciun G, Feinberg M: **Multiple equilibria in complex chemical reaction networks: I. The injectivity property**. *SIAM J. Appl. Math.* 2005, **65**(5):1526–1546.
2. Craciun G, Feinberg M: **Multiple equilibria in complex chemical reaction networks: II. The species-reaction graph**. *SIAM J. Appl. Math.* 2006, **66**(4):1321–1338.
3. Craciun G, Feinberg M: **Multiple equilibria in complex chemical reaction networks: semi-open mass action systems**. *In press* 2009, :1–19.
4. Craciun G, Tang Y, Feinberg M: **Understanding bistability in complex enzyme-driven reaction networks**. *Proc. Natl. Acad. Sci. USA* 2006, **103**(23):8697–8702.

Supplementary Tables

Table S1 - Construction of two-component regulatory subnetworks

Twenty-seven reactions are combined to generate 40,680 subnetworks with two genes and two gene products. The reactions labeled $*$ are included in every subnetwork.

Reaction label	Reaction
$*$	$X_1 \rightarrow X_1 + P_1$
$*$	$X_2 \rightarrow X_2 + P_2$
$*$	$P_1 \rightarrow \emptyset$
$*$	$P_2 \rightarrow \emptyset$
a	$X_1 + P_1 \rightleftharpoons X_1P_1$
b	$X_1 + P_2 \rightleftharpoons X_1P_2$
c	$X_2 + P_1 \rightleftharpoons X_2P_1$
d	$X_2 + P_2 \rightleftharpoons X_2P_2$
e	$X_1P_1 \rightarrow X_1P_1 + P_1$
f	$X_1P_2 \rightarrow X_1P_2 + P_1$
g	$X_2P_1 \rightarrow X_2P_1 + P_2$
h	$X_2P_2 \rightarrow X_2P_2 + P_2$
i	$P_1 + P_1 \rightleftharpoons P_1P_1$
j	$P_1 + P_2 \rightleftharpoons P_1P_2$
k	$P_2 + P_2 \rightleftharpoons P_2P_2$
l	$X_1 + P_1P_1 \rightleftharpoons X_1P_1P_1$
m	$X_1 + P_1P_2 \rightleftharpoons X_1P_1P_2$
n	$X_1 + P_2P_2 \rightleftharpoons X_1P_2P_2$
o	$X_2 + P_1P_1 \rightleftharpoons X_2P_1P_1$
p	$X_2 + P_1P_2 \rightleftharpoons X_2P_1P_2$
q	$X_2 + P_2P_2 \rightleftharpoons X_2P_2P_2$
r	$X_1P_1P_1 \rightarrow X_1P_1P_1 + P_1$
s	$X_1P_1P_2 \rightarrow X_1P_1P_2 + P_1$
t	$X_1P_2P_2 \rightarrow X_1P_2P_2 + P_1$
u	$X_2P_1P_1 \rightarrow X_2P_1P_1 + P_2$
v	$X_2P_1P_2 \rightarrow X_2P_1P_2 + P_2$
w	$X_2P_2P_2 \rightarrow X_2P_2P_2 + P_2$

Table S2 - Two-component regulatory subnetworks analyzed with the CRNT

A subset of small gene regulatory networks containing two TF genes and two gene products analyzed with the CRNT. *Subnetwork* refers to the constituent reactions listed in Table S1; the reactions labeled $*$ in Table S1 are included in every subnetwork analyzed here. The entries *yes* (*no*) indicate that a subnetwork does (does not) contain a reaction of a given type. If *yes*, the number of reactions of that type are given in parentheses. The entries + (−) represents TF-promoter interactions that are productive (unproductive). With the exception of subnetwork *abdefjk*, the CRNT provided example sets of rate constants leading to multiple stable equilibria in subnetworks for which the capacity for multiple equilibria was not ruled out.

Subnetwork	Dimer formation	Monomer binding		Dimer binding		Multiple equilibria ruled out?	
		+	−	+	−	INJ	CRNT
bfijkq	*yes* (3)	*yes* (1)	*no*	*no*	*yes* (1)	*no*	*yes*
acdeijkq	*yes* (3)	*yes* (1)	*yes* (2)	*no*	*yes* (1)	*no*	*yes*
abcdfhjk	*yes* (2)	*yes* (2)	*yes* (2)	*no*	*no*	*no*	*no*
bdhjk	*yes* (2)	*yes* (1)	*yes* (1)	*no*	*no*	*no*	*yes*
adeijkn	*yes* (3)	*yes* (1)	*yes* (1)	*no*	*yes* (1)	*no*	*yes*
bcfgijk	*yes* (3)	*yes* (2)	*no*	*no*	*no*	*no*	*yes*
abdijk	*yes* (3)	*no*	*yes* (3)	*no*	*no*	*no*	*yes*
adijkqw	*yes* (3)	*no*	*yes* (2)	*yes* (1)	*no*	*no*	*no*
acdegjk	*yes* (2)	*yes* (2)	*yes* (1)	*no*	*no*	*no*	*yes*
abcdijk	*yes* (3)	*no*	*yes* (4)	*no*	*no*	*no*	*yes*
adeijk	*yes* (3)	*yes* (1)	*yes* (1)	*no*	*no*	*no*	*yes*
abcdehjk	*yes* (2)	*yes* (2)	*yes* (2)	*no*	*no*	*no*	*no*
bdfhikq	*yes* (2)	*yes* (2)	*yes* (2)	*no*	*yes* (1)	*no*	*yes*
cgk	*yes* (1)	*yes* (1)	*no*	*no*	*no*	*yes*	*yes*
ijk	*yes* (3)	*no*	*no*	*no*	*no*	*yes*	*yes*
acdegjk	*yes* (2)	*yes* (2)	*yes* (1)	*no*	*no*	*no*	*yes*
acdhjk	*yes* (2)	*yes* (1)	*yes* (2)	*no*	*no*	*no*	*yes*
abcejk	*yes* (2)	*yes* (1)	*yes* (2)	*no*	*no*	*no*	*no*
abdefjk	*yes* (2)	*yes* (2)	*yes* (1)	*no*	*no*	*no*	*no*
adijknt	*yes* (3)	*no*	*yes* (2)	*yes* (1)	*no*	*no*	*yes*
bdfhijkn	*yes* (3)	*yes* (2)	*no*	*no*	*yes* (1)	*no*	*yes*
bdfhjk	*yes* (2)	*yes* (2)	*no*	*no*	*no*	*no*	*yes*
abefjk	*yes* (2)	*yes* (2)	*no*	*no*	*no*	*no*	*no*
ikn	*yes* (2)	*no*	*no*	*no*	*yes* (1)	*yes*	*yes*
bcfk	*yes* (1)	*yes* (1)	*yes* (1)	*no*	*no*	*no*	*yes*

Table S3 - Analysis of two-component regulatory subnetworks, excluding those that contain productive transcription factor binding

The percentage of two-component subnetworks, excluding those that contain productive transcription factor binding, for which multiple equilibria can be ruled out using the INJ method is shown. The total number of such subnetworks is reduced to 1030 (from 40,680), and the number of sampled subnetworks is reduced to 542 (from the ∼15,000 analyzed in Table 3). See legend to Table 3.

		Sampled subnetworks	
# dimers binding	% of total models	% of total models	% with multiple equilibria ruled out
0	6.99	5.4 [3.5, 7.2]	72.4 [53.0, 86.7]
1	18.64	16.4 [13.3, 19.6]	55.1 [44.3, 64.9]
2	29.13	29.7 [25.8, 33.4]	37.9 [30.8, 45.6]
3	24.85	25.1 [21.4, 28.8]	37.5 [29.3, 45.5]
4	14.76	16.8 [13.7, 19.9]	22.0 [14.1, 31.5]
5	4.66	5.2 [3.3, 7.0]	7.1 [0.0, 23.8]
6	0.97	1.5 [0.6, 2.6]	0.0

Table S4 - Analysis of two-component regulatory subnetworks, excluding those that contain productive transcription factor binding

The percentage of two-component subnetworks, excluding those that contain productive transcription factor binding, for which multiple equilibria can be ruled out using the INJ method is shown. The total number of such subnetworks is reduced to 1030 (from 40,680), and the number of sampled subnetworks is reduced to 592 (from the ∼15,000 analyzed in Table 3). Compare Table S3.

		Sampled subnetworks	
# dimers binding	% of total models	% of total models	% with multiple equilibria ruled out
0	6.99	5.4 [3.7, 7.3]	25.0 [11.1, 41.9]
1	18.64	15.2 [12.3, 18.1]	21.1 [13.6, 30.7]
2	29.13	30.4 [26.5, 34.0]	12.8 [8.4, 18.3]
3	24.85	24.8 [21.3, 28.2]	6.8 [3.4, 11.9]
4	14.76	18.2 [15.0, 21.3]	1.9 [0.0, 6.4]
5	4.66	4.9 [3.2, 6.8]	0.0
6	0.97	1.0 [0.3, 1.9]	0.0

Supplementary Figures

Figure S1. SR graph theorem analysis for network subnetworks abcdefg, abcdef, abcefg, and abcef

In the reaction networks shown here (*abcdefg*, *abcdef*, *abcefg*, and *abcef*), multiple equilibria cannot be ruled out by the SRG method based on the species-reaction graph of the CRN [4]. In each of these networks there exists a cycle (highlighted in yellow) that is both an even cycle and not a 1-cycle; the arc from the species P the reaction $\mathrm{P} + \mathrm{P} \leftrightarrow \mathrm{PP}$ has a stoichiometric coefficient of two. Thus, the first property of the SR graph theorem does not hold for these networks.

Figure S2. SR graph theorem analysis for network subnetworks abefg and abef

Similar to those networks shown in Fig. S1, multiple equilibria cannot be ruled out by the SR graph theorem in the reaction networks shown here (*abefg* and *abef*) because the first property of the SR graph theorem does not hold. As before, in each of these networks there exists a cycle (highlighted in pink) that is both an even cycle and not a 1-cycle; the arc from the species P the reaction $\mathrm{P} + \mathrm{P} \leftrightarrow \mathrm{PP}$ has a stoichiometric coefficient of two.

Figure S3. SR graph theorem analysis for network subnetworks abcde, abcd, abc, and abce

The SR graph theorem does not rule out multiple equilibria for the networks shown above (*abcde*, *abcd*, *abc*, and *abce*). In all cases, the two even cycles highlighted (in blue and tan) split the $\mathrm{X} + \mathrm{P}$ c-pair, and therefore do not satisfy the second property of the theorem.

Figure S4. SR graph theorem analysis for network subnetworks ab and abe

Bistability is ruled out by the SR graph theorem for the simple networks shown here (*ab* and *abe*). In both networks, there is only a single 1-cycle (indicated in blue), and thus there are no split c-pairs. Both of the network properties required by the theorem to rule out bistability are satisfied.

Figure S5. Bifurcation diagrams for networks abcefg and abcdefg

The networks *abcefg* and *abcdefg* were shown by the CRNT to each admit two stable equilibria, as can be seen in these bifurcation diagrams. Stable steady states are denoted with solid lines, and unstable steady states are denoted with dashed lines. Equilibrium concentrations for X, P, PP, XPP, and XP are plotted as a function of the association rate constant for dimer formation. For network $abcefg$, the parameters obtained from the CRNT are: $k_{\mathrm{X}\to\mathrm{X+P}} = 3.26$, $k_{\mathrm{X+P}\to\mathrm{XP}} = 0.10$, $k_{\mathrm{P}\to\emptyset} = 1$, $k_{\mathrm{XP}\to\mathrm{X+P}} = 2.40$, $k_{\mathrm{PP}\to\mathrm{P+P}} = 0.98$, $k_{\mathrm{X+PP}\to\mathrm{XPP}} = 3.20$, $k_{\mathrm{XPP}\to\mathrm{X+PP}} = 3.61$, and $k_{\mathrm{XPP}\to\mathrm{XPP+P}} = 109.0$. For network

$abcdefg$, the parameters obtained from the CRNT are: $k_{\mathrm{X}\to\mathrm{X+P}} = 6.16$, $k_{\mathrm{X+P}\to\mathrm{XP}} = 0.13$, $k_{\mathrm{P}\to\emptyset} = 1$, $k_{\mathrm{XP}\to\mathrm{X+P}} = 21.70$, $k_{\mathrm{XP}\to\mathrm{XP+P}} = 21.70$, $k_{\mathrm{PP}\to\mathrm{P+P}} = 402.4$, $k_{\mathrm{X+PP}\to\mathrm{XPP}} = 422.8$, $k_{\mathrm{XPP}\to\mathrm{X+PP}} = 479.8$, and $k_{\mathrm{XPP}\to\mathrm{XPP+P}} = 479.8$.

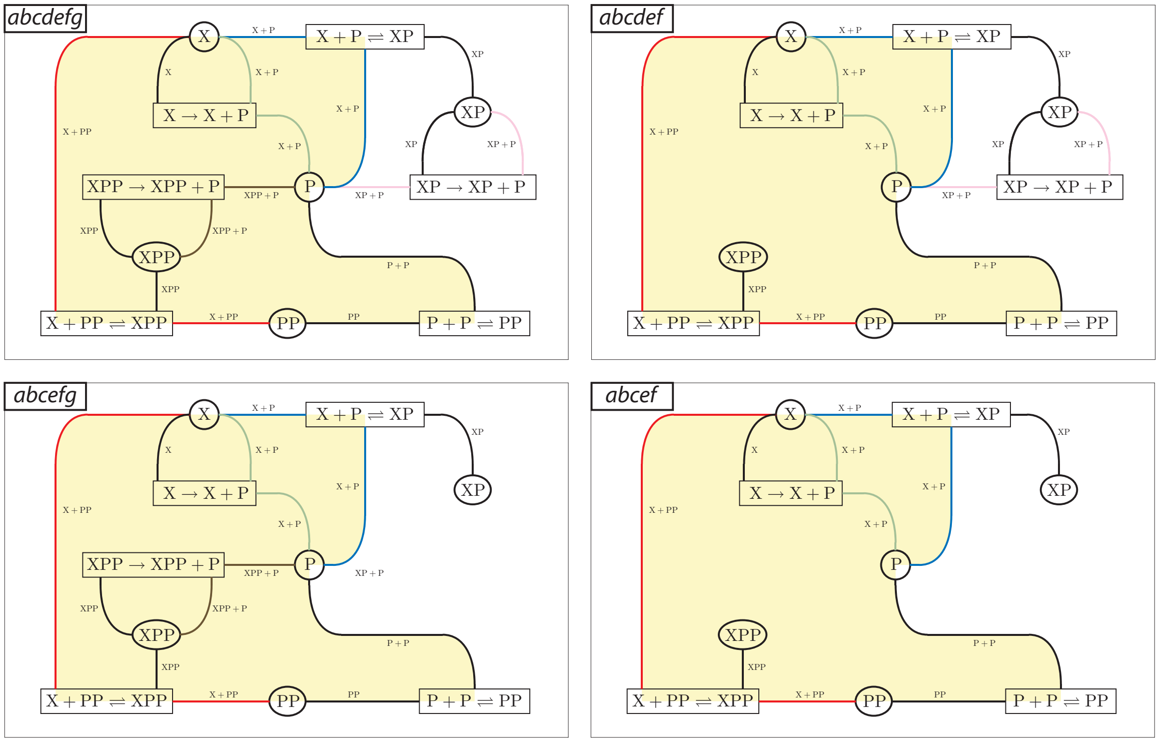

Figure S1: SR graph theorem analysis for network subnetworks *abcdefg*, *abcdef*, *abcefg*, and *abcef*

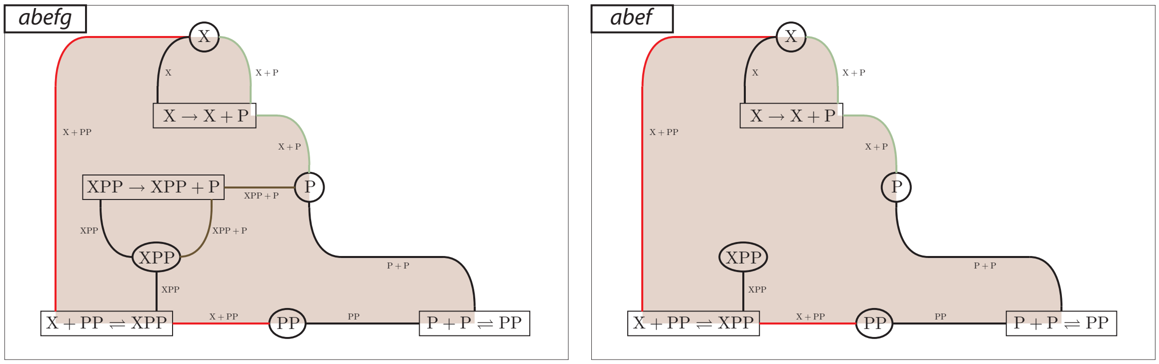

Figure S2: SR graph theorem analysis for network subnetworks *abefg* and *abef*

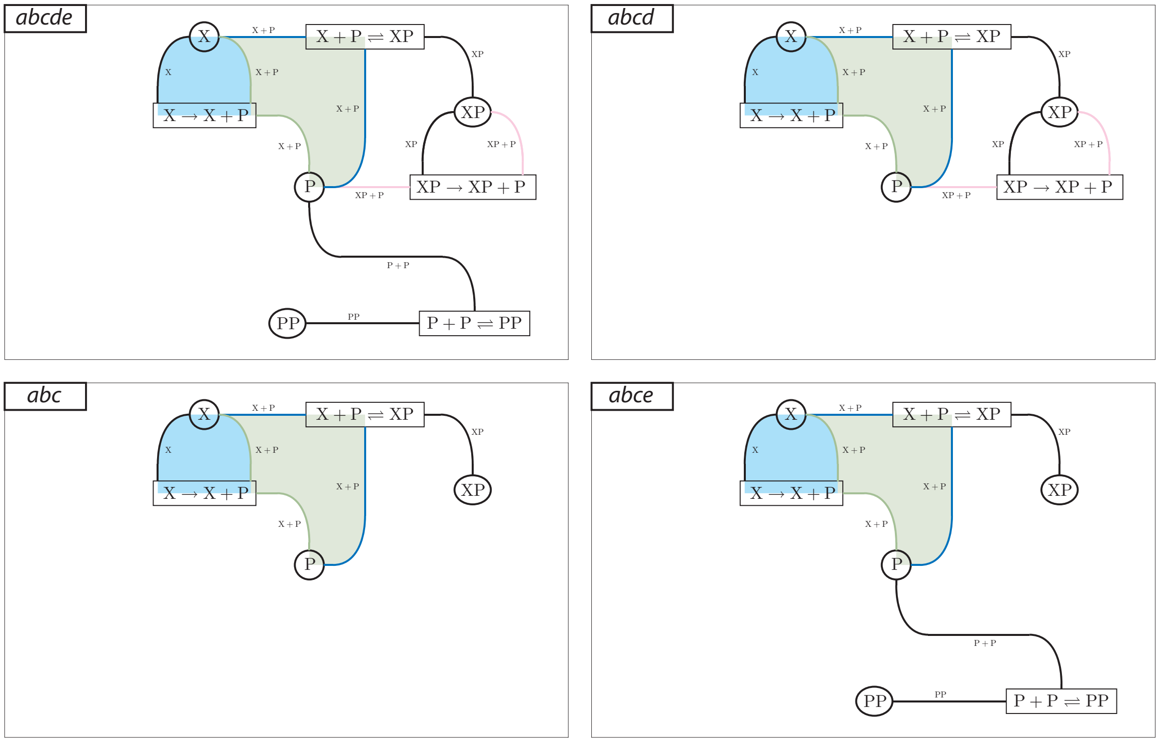

Figure S3: SR graph theorem analysis for network subnetworks *abcde*, *abcd*, *abc*, and *abce*

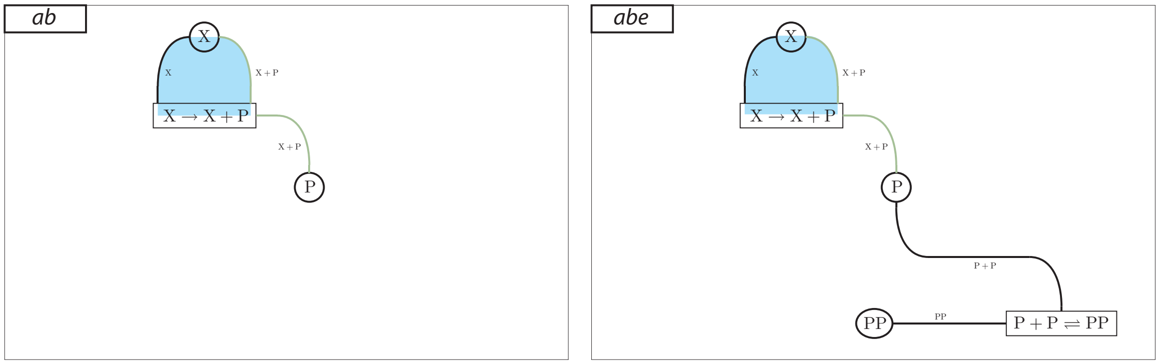

Figure S4: SR graph theorem analysis for network subnetworks *ab* and *abe*

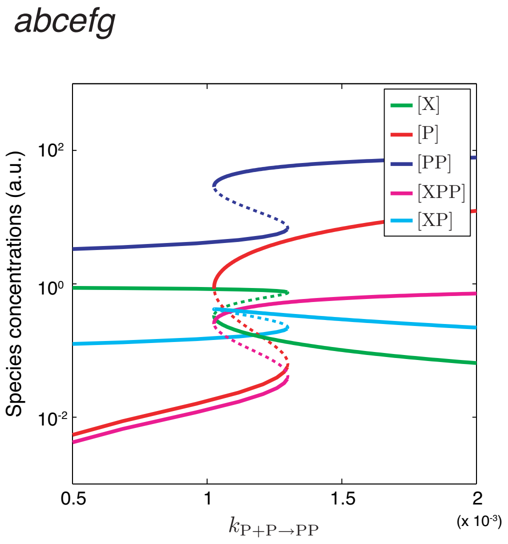

Figure S5: Bifurcation diagrams for subnetworks *abcefg* and *abcdefg*
